# Supplementary material for: The structure and statistics of language jointly shape cross-frequency neural dynamics during spoken language comprehension
Source: Nat Commun. 2024 Oct 14;15:8850. doi: 10.1038/s41467-024-53128-1 (PMC11471778; doi:10.1038/s41467-024-53128-1)
Supplement: Supplementary file 3 — Reporting Summary [file 41467_2024_53128_MOESM3_ESM.pdf]

nature portfolio

Corresponding author(s): Hugo Weissbart

Last updated by author(s): Jul 25, 2024

Reporting Summary

Nature Portfolio wishes to improve the reproducibility of the work that we publish. This form provides structure for consistency and transparency in reporting. For further information on Nature Portfolio policies, see our Editorial Policies and the Editorial Policy Checklist.

Statistics

For all statistical analyses, confirm that the following items are present in the figure legend, table legend, main text, or Methods section.

n/a	Confirmed	
☐	☒	The exact sample size (*n*) for each experimental group/condition, given as a discrete number and unit of measurement
☐	☒	A statement on whether measurements were taken from distinct samples or whether the same sample was measured repeatedly
☐	☒	The statistical test(s) used AND whether they are one- or two-sided<br>*Only common tests should be described solely by name; describe more complex techniques in the Methods section.*
☐	☒	A description of all covariates tested
☐	☒	A description of any assumptions or corrections, such as tests of normality and adjustment for multiple comparisons
☐	☒	A full description of the statistical parameters including central tendency (e.g. means) or other basic estimates (e.g. regression coefficient) AND variation (e.g. standard deviation) or associated estimates of uncertainty (e.g. confidence intervals)
☐	☒	For null hypothesis testing, the test statistic (e.g. *F*, *t*, *r*) with confidence intervals, effect sizes, degrees of freedom and *P* value noted<br>*Give P values as exact values whenever suitable.*
☒	☐	For Bayesian analysis, information on the choice of priors and Markov chain Monte Carlo settings
☒	☐	For hierarchical and complex designs, identification of the appropriate level for tests and full reporting of outcomes
☒	☐	Estimates of effect sizes (e.g. Cohen's *d*, Pearson's *r*), indicating how they were calculated

Our web collection on statistics for biologists contains articles on many of the points above.

Software and code

Policy information about availability of computer code

Data collection	MEG data was collected using a 275-channel axial gradiometer CTF system in magnetically shielded room. While recording, visual and auditory stimuli were presented using Psychtoolbox for MATLAB Version 9.4 (R2018a). Structural MRI data was collected using a 3T MAGNETOM Skyra MR scanner (Siemens AG) at the Donders Centre for Cognitive Neuroimaging in Nijmegen.
Data analysis	The code supporting the findings of this study is available on the GitHub repository at https://github.com/Hugo-W/feature-PAC. All the analysis was carried out using Python 3.12 using custom code, using MNE-Python library (version 1.6.1) and scientific packages (numpy version 1.26.4, scipy version 1.13.0). Figures are done using Matplotlib (version 3.8.3) and Seaborn (version 0.13.2).

For manuscripts utilizing custom algorithms or software that are central to the research but not yet described in published literature, software must be made available to editors and reviewers. We strongly encourage code deposition in a community repository (e.g. GitHub). See the Nature Portfolio guidelines for submitting code & software for further information.

Data

Policy information about availability of data

All manuscripts must include a data availability statement. This statement should provide the following information, where applicable:

- Accession codes, unique identifiers, or web links for publicly available datasets
- A description of any restrictions on data availability
- For clinical datasets or third party data, please ensure that the statement adheres to our policy

The raw MEG data generated in this study have been deposited in the Radboud University Repository database and are openly available with the identifier

doi.org/10.34973/a65x-p009. Processed MEG data and Source data underlying the figures in this paper are available in Figshare with the identifier doi.org/10.6084/m9.figshare.24236512.

Research involving human participants, their data, or biological material

Policy information about studies with human participants or human data. See also policy information about sex, gender (identity/presentation), and sexual orientation and race, ethnicity and racism.

Reporting on sex and gender	Gender information from participants were included based on self-reported gender. The final sample for the MEG experiment included 18 women and 7 men. No gender-based analyses were performed.
Reporting on race, ethnicity, or other socially relevant groupings	No racial or ethnic information was collected.
Population characteristics	All participants were educated to degree level or above with no self-reported history of neurological or psychiatric illness or hearing impairment. For the sample included in the MEG experiment the age range was 18 to 58 years old, all right handed.
Recruitment	Participants were recruited via posters placed in public areas (e.g. university) and suitable websites (e.g., university websites). Notably the university of Radboud holds a website and keep a participant database especially for recruiting participants.
Ethics oversight	The study was approved by the ethical commission for human research in Arnhem and Nijmegen (CMO2014/288). Informed consent was obtained from every participant. Participants were given monetary reimbursement for their participation.

Note that full information on the approval of the study protocol must also be provided in the manuscript.

Field-specific reporting

Please select the one below that is the best fit for your research. If you are not sure, read the appropriate sections before making your selection.

☐ Life sciences ☒ Behavioural & social sciences ☐ Ecological, evolutionary & environmental sciences

For a reference copy of the document with all sections, see nature.com/documents/nr-reporting-summary-flat.pdf

Behavioural & social sciences study design

All studies must disclose on these points even when the disclosure is negative.

Study description	The MEG experiment consisted of continuous recording during listening of audiobook stories. Behavioural assessment of performance were also taken throughout the experiment.
Research sample	A total of 25 participants (18 women, between 18 and 58 years old) completed the experiment. All participants were right-handed native Dutch speakers with no reported fluency in French despite incidental exposure. Participants self-reported their (in)ability to understand a sentence in French. Participants were reporting no history of hearing or language impairment.
Sampling strategy	Sample sizes of approximately 30 participants have shown to be reliable to detect significant effects in similar experimental designs with 80% power at p = 0.05 significance level.
Data collection	MEG data were recorded using a 275-channel CTF system. Headhsape was measured using a Polhemus Isotrak system. Finally, structural MRI scan (T1-weighted) were taken using a 3T MAGNETOM Skyra system. Two researchers of our group were present during each recording session.
Timing	The collection took place between December 2020 until June 2022, with a slower pace in the first part of the recruitment because of new regulations about bringing human participants during the COVID19 pandemic.
Data exclusions	One of the initially recruited participant data were left out because of metal artifacts visible on the MEG data, and another could not complete the entire experiment because of technical issues.
Non-participation	No participant dropped out nor declined.
Randomization	The order of the stimulus presentation were randomized across subjects.

Reporting for specific materials, systems and methods

We require information from authors about some types of materials, experimental systems and methods used in many studies. Here, indicate whether each material, system or method listed is relevant to your study. If you are not sure if a list item applies to your research, read the appropriate section before selecting a response.

Materials & experimental systems

n/a	Involved in the study
☒	☐ Antibodies
☒	☐ Eukaryotic cell lines
☒	☐ Palaeontology and archaeology
☒	☐ Animals and other organisms
☒	☐ Clinical data
☒	☐ Dual use research of concern
☒	☐ Plants

Methods

n/a	Involved in the study
☒	☐ ChIP-seq
☒	☐ Flow cytometry
☐	☒ MRI-based neuroimaging

Plants

Seed stocks

Report on the source of all seed stocks or other plant material used. If applicable, state the seed stock centre and catalogue number. If plant specimens were collected from the field, describe the collection location, date and sampling procedures.

Novel plant genotypes

Describe the methods by which all novel plant genotypes were produced. This includes those generated by transgenic approaches, gene editing, chemical/radiation-based mutagenesis and hybridization. For transgenic lines, describe the transformation method, the number of independent lines analyzed and the generation upon which experiments were performed. For gene-edited lines, describe the editor used, the endogenous sequence targeted for editing, the targeting guide RNA sequence (if applicable) and how the editor was applied.

Authentication

Describe any authentication procedures for each seed stock used or novel genotype generated. Describe any experiments used to assess the effect of a mutation and, where applicable, how potential secondary effects (e.g. second site T-DNA insertions, mosiacism, off-target gene editing) were examined.

Magnetic resonance imaging

Experimental design

Design type

The MRI scanner was used only to acquire structural images, no task.

Design specifications

N/A

Behavioral performance measures

N/A

Acquisition

Imaging type(s)

T1-weighted

Field strength

3T

Sequence & imaging parameters

Magnetization-prepared rapid gradient-echo (MP-RAGE) pulse sequence was used, parameters: volume TR=2300ms, TE=3.03ms, 8 degree flip-angle, 1 slab, slice-matrix size=256×256, slice thickness=1mm, field of view=256mm, isotropic voxel-size=1.0×1.0×1.0mm

Area of acquisition

Whole brain scans

Diffusion MRI ☐ Used ☒ Not used

Preprocessing

Preprocessing software

MNE-python (version 1.6.1) was used to import and preprocessed MEG data and FreeSurfer (freesurfer-linux-centos7_x86_64-7.3.2-20220804-6354275) were used for processing MRI structural images.

Normalization

Source reconstructed data were morphed onto source model for fsaverage in MNI305 coordinate.

Normalization template

Coordinate used for normalisation and mapping to FreeSurfer's fsaverage brain are done in MNI305 coordinate, done using MNE-python.

Noise and artifact removal

N/A

Volume censoring

N/A

Statistical modeling & inference

Model type and settings

MRI data were not used for analysis

Effect(s) tested	N/A

Specify type of analysis: ☐ Whole brain ☐ ROI-based ☐ Both

Statistic type for inference (See Eklund et al. 2016)	*Specify voxel-wise or cluster-wise and report all relevant parameters for cluster-wise methods.*
Correction	*Describe the type of correction and how it is obtained for multiple comparisons (e.g. FWE, FDR, permutation or Monte Carlo).*

Models & analysis

n/a	Involved in the study
☒	☐ Functional and/or effective connectivity
☒	☐ Graph analysis
☒	☐ Multivariate modeling or predictive analysis
